# Attrition from Care and Clinical Outcomes in a Cohort of Sickle Cell Disease Patients in a Tribal Area of Western India

**DOI:** 10.3390/tropicalmed4040125

**Published:** 2019-10-01

**Authors:** Kapilkumar Dave, Palanivel Chinnakali, Pruthu Thekkur, Shrey Desai, Chandrakant Vora, Gayatri Desai

**Affiliations:** 1 Society for Education Welfare and Action Rural, Jhagadia, Gujarat Pin-393110, India; sdesai1977@yahoo.com (S.D.); ckvora@yahoo.com (C.V.); shreygayatri@gmail.com (G.D.); 2 Department of Preventive and Social Medicine, Jawaharlal Institute of Postgraduate Medical Education and Research (JIPMER), Puducherry Pin-605006, India; palaniccm@gmail.com; 3 Monitoring and Evaluation Officer, Centre for Operational Research, International Union Against Tuberculosis and Lung Disease, Paris Pin-75006, France; pruthu.TK@theunion.org; 4 Monitoring and Evaluation Officer, Centre for Operational Research, The Union South-East Asia Office, New Delhi Pin-110016, India

**Keywords:** disadvantaged population, inherited disease, lost to follow up, sickle cell disease program

## Abstract

In a tribal area of western India, a non-governmental organization implemented a comprehensive sickle cell disease (SCD) program at a secondary level hospital. In a cohort of SCD patients registered during December 2015 to June 2017, we assessed rates of lost to follow-up (LTFU) during the follow-up period using routinely collected data. We compared the uptake of proven interventions and indicators of disease severity from one year prior to registration until the end of the study (June 2018). Of 404 patients, the total follow-up duration was 534 person-years (PY). The rate (95% CI) of LTFU was 21 (17.5–25.3) per 100 PY. The proportion of people who received the pneumococcal vaccine improved from 10% to 93%, and coverage of hydroxyurea improved from 3.5% to 88%. There was a statistically significant decrease in rates (per 100 PY) of pain crisis (277 vs 53.4), hospitalization (49.8 vs 42.2), and blood transfusion (27.4 vs 17.8) after enrollment in the SCD program. Although clinical intervention uptake was high, one quarter of the patients were LTFU. The study demonstrated significant reductions in disease severity in SCD patients.

## 1. Introduction

Sickle cell disease (SCD) is a common genetic condition characterized by the tendency of red blood cells to become sickle-shaped and block capillaries, leading to vaso-occlusion and anemia. In children, sickle-shaped red blood cells often become trapped in the spleen, leading to a serious risk of death before the age of seven years from a sudden profound anemia or due to an overwhelming infection [1]. Thus, SCD remains an important contributor of childhood mortality and premature death in adult population [2,3,4]. SCD is more common among tribal communities than the general population [5,6]. In India, tribal populations constitute 8.6% of the total Indian population, about 68 million people [7]. The prevalence of sickle cell heterozygous (SCD trait) and SCD varies, with rates of about 1–40% and 1–12%, respectively, in Indian tribal populations [5,6,8].

As the burden of SCD is high among tribal populations who predominantly inhabit hilly terrain, delivering healthcare to these groups remains a challenge [8]. The current clinical management of patients suffering from sickle cell disease in India is inadequate, and basic facilities to manage patients are usually absent or sub-optimal even in tribal pockets where the burden of SCD is high [9]. The World Health Organization (WHO) in its 2006 report recommended that, in areas where SCD is common, dedicated centers should be erected to ensure adequate services for prevention and treatment. However, a model of a national control program developed in high-resource countries could not be replicated in low-resource settings [1]. The effectiveness of interventions, including pneumococcal vaccination, newborn screening, and penicillin prophylaxis, has been well studied in Western countries.

In India, during the last decade, sickle cell disease programs were started under the National Rural Health Mission in selected high-burden districts [10,11]. Activities under the program include mass screening of tribal groups and provision of care using public health facilities [12]. Although screening was undertaken, the availability and coverage of proven interventions, like prophylactic and therapeutic hydroxyurea and pneumococcal vaccination, were sub-optimal in public health facilities due to various reasons, for example, challenges relating to access to tribal areas [9,13].

Since 2014, in Bharuch district of Gujarat, India, a comprehensive SCD program was provided at Kasturba hospital, a secondary care hospital under the Society for Education Welfare and Action (SEWA) Rural, a non-governmental organization (NGO). Proven interventions for SCD were provided for free or at a subsidized cost through sickle cell clinics. However, there is a need to audit the process of care and outcome indicators of this model [11]. Therefore, we aimed to describe the management and treatment outcomes in a cohort of sickle cell disease patients registered with an NGO-led, comprehensive sickle cell disease program in a tribal area of Gujarat. The specific objectives of the study were (a) to describe the socio-demographic and clinical profiles of patients registered with sickle cell disease, (b) to determine the time taken until patients became lost to follow-up and the associated factors, (c) to determine the proportion of patients receiving clinical interventions as per the standard treatment guidelines, and (d) to describe the clinical outcomes, including number of hospitalization episodes, number of pain crises, number of blood transfusions, rate of severe anemia, and complications due to sickle cell disease at different time points.

## 2. Material and Methods

### 2.1. Study Design and Setting

This was a longitudinal descriptive study using routinely collected data at Kasturba hospital, a secondary level hospital in the Bharuch district in southern Gujarat, India. The 200 bed hospital is run by SEWA Rural, catering to about 1500 villages with a predominantly tribal population. Both outpatient and inpatient services are provided free of charge for 70–80% of patients (based on self-reported income), with others receiving subsidized care. The hospital acts as the first referral unit for maternal and child health (MCH) services. 

In February 2014, a comprehensive sickle cell program was started at the hospital to provide continuity of care to patients with sickle cell disease. The care model included screening, standardized outpatient and inpatient protocols, health education, counseling, and information and technology (IT) applications for monitoring and follow-up of these patients. All pregnant women, newborns of mothers with sickle cell trait or disease, family members of patients with sickle cell disease, and patients with anemia or symptoms of sickle cell disease were screened for sickle cell disease. Those screened positive were enrolled in the program and were followed up once every three months in the sickle cell clinic. The hospitals also provided emergency and inpatients services for the management of complications of the disease, including pain crisis. A blood storage unit was located at the hospital and blood transfusion was done for patients with severe anemia (<7.0 gm/dL). 

The 23-valent pneumococcal polysaccharide vaccine (PPV23) was provided to all sickle cell disease patients aged more than two years. Patients were provided daily doses of folic acid. Patients who experienced at least 3 episodes of pain crisis or 3 hospitalizations due to sickle cell disease or 3 blood transfusions within the last year were marked as severe sickle cell disease patients. All severe sickle cell disease patients were provided with hydroxyurea medicine. A complete hemogram, renal function tests, and liver function tests were performed annually. A web-based management information system (MIS) captured the above information at baseline and also during follow-up visits every three months. Hospitalization data were updated in MIS by a dedicated counselor who visited the patients during their admission. Details of hospitalization in other health facilities and incidences of pain crisis were collected during the follow-up visits and updated in MIS. 

The counselor educated patients during visits to the sickle cell clinic about the disease, its complications, including pain crisis, and adherence to the care. If any patient missed the scheduled visit, MIS sent an alert to the counselor for retrieval action. The counselor made telephone calls to the patients to retrieve them back to care. Deaths were confirmed with patient’s relatives through telephone calls. Data entry and completeness were assessed once a week by medical officer. A review of the program was done once a month, with reports generated by MIS. 

### 2.2. Study Population

We included all patients who were registered in a comprehensive sickle cell disease program from December 2015 to June 2017. 

### 2.3. Data Variables and Data Sources

Variables included socio-demographic characteristics, lost to follow-up for care, death, hemoglobin levels at registration and during follow-up visits, number of pain crisis episodes, hospitalization, blood transfusions from one year before registration and during follow-up visits, and status of proven interventions, like the pneumococcal vaccine and hydroxyurea therapy, at the time of registration and thereafter, which were confirmed by interviewing patients during hospital visits. Tests included a complete hemogram, renal function tests, and liver function tests during follow-up. Information on the above variables was extracted from MIS in July 2018. De-identified data without patient names were used for analysis. As this study presented analyses of secondary, programmatic, routinely collected data, participant consent was not obtained, although hospital permission was sought before extracting the information. The dataset is available on https://www.dropbox.com/sh/cb7uawqslw9kmeu/AADtk2zqj0rm9ceUsLZcGWkta?dl=0.

### 2.4. Data Analysis

Data extracted in Microsoft Excel was analyzed using EpiData analysis software version 2.2.2.186 (EpiData association, Odense, Denmark) and Stata analysis software version 14. 

Socio-demographic and clinical characteristics were summarized as proportions. We defined lost to follow-up (LTFU) as missing two consecutive scheduled visits to the sickle cell clinic. We performed time-to-event analysis to assess the timing of LTFU during care. The censoring date was taken as either the date of death or 30 June 2018. To assess the possible associations of socio-demographic and clinical characteristics with LTFU, we estimated unadjusted and adjusted hazard ratios with 95% confidence intervals using the Cox proportional hazards model. We included variables with p values less than 0.2 for adjusted analysis and p values less than 0.05 were considered to be statistically significant. We estimated the aerial distance between patients’ residences and the hospital using the distance matrix application in QGIS software version 2.18.15, and these data were included in the analysis.

We calculated rates per 100 person-years of clinical outcomes (hospitalization, pain crisis, and blood transfusion) for two time periods, namely, one year before registration and after registration to 30 June 2018. Intervention coverage (pneumococcal vaccine, hydroxyurea, and folic acid supplementation) and investigations (renal function test, liver function test, and complete blood counts) were presented as proportions. 

### 2.5. Ethical Approval

Ethics approval was obtained from the Ethics Advisory Group of The Union, Paris, France (EAG No: 18/18, 12 April 2018) and SEWA Rural institutional ethics committee (SR/IEC/2018/05/1, 26 May 2018). Permission and support for the operation research (OR) were sought from the management of the Kasturba hospital, SEWA Rural, before initiating the OR.

## 3. Results

**Figure 1:**
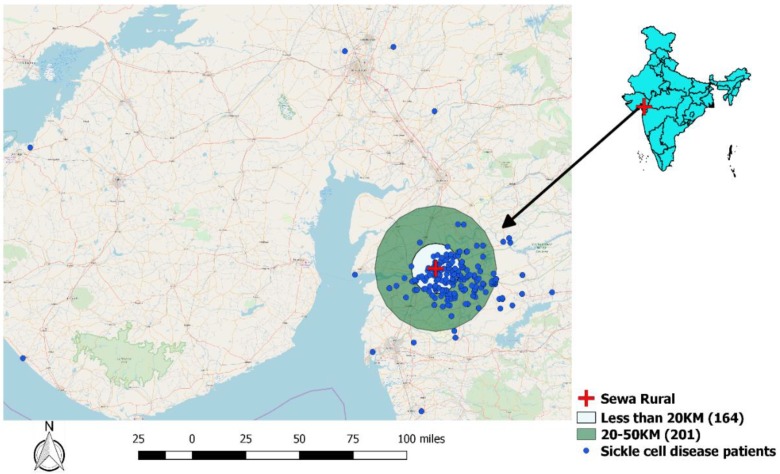
Map depicting the distribution of sickle cell disease patients registered for care in SEWA Rural.

A total of 404 SCD patients were registered during the reference period, including 247 (61%) females with a mean (SD) age of 19 (9) years. Children (0–14 years) constituted 26% of total patients and about 11% did not have formal education. Of the 404 patients, 401 (99%) were from a tribal community. Socio-demographic characteristics of study participants are described in Table 1. Of the 404 patients, 365 (90.3%) had a functional mobile number documented with the SCD program. The geographical distribution of the SCD patients and the distance from the study hospital are shown in Figure 1. About 90% of the patients resided within a radius of 50 kilometers from the hospital.

Regarding the mode of enrollment into the SCD program, 113 (28%) patients were inpatients at the time of registration and the other 291 (72%) patients were enrolled from an outpatient clinic. The number and proportion of patients who had pain crisis, hospitalization, and blood transfusion one year prior to registration were 356 (88%), 131 (32%), and 72 (18%) respectively. The genotype results were available for 201 (49%) participants. Of the 201 patients, 195 (97%) had the sickle hemoglobin (HBS)-homozygous genotype. The clinical characteristics of the SCD patients are described in Table 2.

The total follow-up duration was 534 person-years (PY) and the median (IQR) follow-up period was 16.8 (9.5–23.5) months. In total, 112 (28%) patients were LTFU during the follow-up period. The rate of LTFU was 21 per 100 PY (95% CI: 17.5–25.3). The associations between the baseline socio-demographic characteristics and clinical characteristics one year prior to registration and LTFU are described in Table 3 and Table 4. LTFU was higher among females than males (25.1 vs 14.9 per 100 PY). In the adjusted analysis, females, patients with no formal education, patients who were hospitalized at the time of registration, and those who had an episode of pain crisis or blood transfusion one year prior to registration had statistically significant higher LTFU rates. 

The differences in coverage of clinical interventions and clinical outcomes between registration and attrition from care or end of the study (30 June 2018) among patients who were registered in a comprehensive care program (N = 404) are described in Table 5. The proportion of patients who received PPV23 improved from 10% to 93%. Similarly, among patients who were eligible for prophylactic or therapeutic hydroxyurea (N = 137), 98.5% patients were initiated on hydroxyurea compared to 4.4% at baseline. The proportion of patients with sickle cell complications increased from 4% to 7.7%. There were statistically significant decreases in the rates of pain crisis (277 vs 53.4 per 100 PY), hospitalization (49.8 vs 42.2 per 100 PY), and blood transfusion (27.4 vs 17.8 per 100 PY) after enrollment in the SCD program.

## 4. Discussion

In this study, we described LTFU and improvement in clinical interventions and outcomes in a comprehensive SCD program run by an NGO in a tribal block. One quarter of the patients enrolled in care were lost to follow-up. There was a high uptake of preventive vaccination and disease modifying therapy. The study demonstrated significant reductions in disease severity assessed in terms of episodes of pain crisis, hospitalization, and blood transfusion.

Our study had a few limitations. Our analysis was based on secondary data routinely collected in the SCD program. We did not have reasons for LTFU as it was not documented routinely. As the frequency of visits to the sickle cell clinic was once every three months, recall bias when reporting events that occurred between visits cannot be ruled out. One of the major limitations was the differential way of ascertaining clinical interventions and outcomes at two time points; at the baseline, we relied mainly on self-reporting by patients, but at the end of the study, we assessed outcomes based on our records. This may have caused an overestimation regarding the improvement in coverage of interventions and clinical outcomes. The study included patients from a secondary care hospital run by an NGO, hence, findings from this study cannot be extrapolated to patients in the public sector. As this was a retrospective study using routinely collected hospital data, information about High Performance liquid chromatography (HPLC) results was not available for all patients. Although we made a prompt attempt to extract these details from the HPLC machine, data deletion occurred for about 50% of patients. Hence, we failed to describe the genotype of all of the patients. 

The LTFU rate in our setting was about 28%. A study from the same setting in 2014 reported a LTFU rate of 12% in a cohort of SCD patients followed up for one year [9]. The higher LTFU rates were due to improvement in the LTFU tracking system with an IT-based background. Higher rates (36%) of LTFU in SCD patients were reported from Gudalur, Tamil Nadu [14]. In our study, LTFU was more likely among those without any formal education. Though not statistically significant in the adjusted analysis, education was an important determinant in seeking healthcare and continuation of care. Qualitative research could explore the reasons for higher LTFU in this sub-group. Although our program had mechanisms to identify patients who were LTFU and a dedicated counselor to track them, these measures may not be sufficient. Adopting good practices from other models of care is needed. A village-based model in Gujarat, using a mobile clinical unit and a local villager who visits and monitors sickle cell disease patients, should be looked at. Similarly, a comprehensive SCD program in developed Tamil Nadu community networks among tribal groups should be investigated to facilitate running clinics in difficult-to-reach areas [14]. 

Our study demonstrated a substantial improvement in the uptake of vaccination and other interventions and a decrease in disease severity. Possible reasons for these results include the comprehensiveness of the SCD program, which had a dedicated team to screen in antenatal clinics and general outpatient clinics, facilities to manage complications, availability of proven interventions for free or at a subsidized cost, and computerized MIS-generated alerts and monthly reports for monitoring purposes. Although NGOs in their capacity build these models of care, efforts into the expansion of these types of models into larger national programs including high-priority inherited diseases is lacking in India. Although state governments have taken initiative in terms of screening and identifying at-risk individuals, sustained commitment regarding chronic disease management is lacking.

Compared to models of care in high-resource settings, our model is predominantly a facility-based one. More decentralized care at the level of primary health centers or at block levels with community support will help to reduce LTFU and improve health outcomes. In high-resource settings, due to better availability of expertise with standard evidence-based guidelines, standard care is available at every level; however, in low-resource settings, resources and expertise are limited and suffer from a lack of standard guidelines, meaning the care models implemented in high-income countries cannot be implemented in low-income countries like India.

Our study has the following strengths. First, we confirmed the deaths of the patients which occurred outside the study hospital by contacting them by mobile calls. This reduced some misclassification between LTFU and death. Second, all information related to patient care was collected routinely using computers or tablets in real time and maintained in MIS. This reduced the number of missing observations and also errors related to data entry and data extraction from paper-based registers. Third, we performed time-to-event analysis to assess the LTFU and death rate, which is appropriate for differential follow-up periods in longitudinal studies. 

## 5. Conclusions

This study demonstrated that good quality, focused care can be delivered to economically disadvantaged and remote populations for a neglected disease like SCD. By making the proven interventions available and accessible to SCD patients, the severity of the disease and hospitalization rates can be reduced. 

## Figures and Tables

**Table 1:** Socio-demographic profiles of patients registered in a comprehensive sickle cell disease program led by a non-governmental organization (NGO) in a tribal area of Gujarat, India during December 2015–June 2017; N = 404.

Characteristics	Categories	n	(%)
Age in years	0–13	105	(26.0)
	14–29	246	(60.9)
	>30	53	(13.1)
Gender	Male	157	(38.9)
	Female	247	(61.1)
Education *	No formal education	45	(11.1)
	Primary	197	(48.8)
	Secondary and above	162	(40.1)
Occupation	Unemployed	172	(42.6)
	Employed	75	(18.6)
	Student	157	(38.9)
Marital Status	Married	168	(41.6)
	Unmarried	236	(58.4)
Caste	Scheduled tribe	401	(99.3)
	Backward classes	3	(0.7)
Aerial distance of house address from SEWA Rural * in KM **	<=20	164	(40.6)
	20–50	201	(49.8)
	>=51	39	(9.7)

* Society for Education Welfare and Action- Rural. ** Kasturba Maternity Hospital.

**Table 2:** Baseline clinical profile of the patients registered in a comprehensive sickle cell disease program led by an NGO in a tribal area of Gujarat, India during December 2015–June 2017; N = 404.

Characteristics	Categories	Children, N = 105		Adults, N = 299		Total	
		n	(%)	n	(%)	n	(%)
Hospitalized at registration	Yes	81	(77.1)	210	(70.2)	113	(28.0)
	No	24	(22.9)	89	(28.8)	291	(72.0)
Number of pain crisis episodes in the last year	0	23	(21.9)	25	(8.4)	48	(11.9)
	1	21	(20.0)	42	(14.0)	63	(15.6)
	2	20	(19.1)	72	(24.1)	92	(22.8)
	>=3	41	(39.0)	160	(53.5)	201	(49.8)
Number of hospitalizations in the last year	0	76	(72.4)	197	(65.9)	273	(67.6)
	1	18	(17.1)	66	(22.1)	84	(20.8)
	2	6	(5.7)	22	(7.3)	28	(6.9)
	>=3	5	(4.8)	14	(4.7)	19	(4.7)
Number of blood transfusions in the last year	0	89	(84.7)	243	(81.3)	332	(82.2)
	1	9	(8.6)	30	(10.0)	39	(9.7)
	>=2	7	(6.7)	26	(8.7)	33	(8.2)
Severe anemia	Yes	24	(22.9)	37	(12.4)	61	(15.1)
	No	81	(87.1)	262	(87.6)	343	(84.9)
Presence of sickle cell complications	Yes	1	(1.0)	15	(5.0)	16	(4.0)
	No	104	(99.0)	284	(95.0)	388	(96.0)
Genotype	HBS *- homozygous	57	(54.3)	138	(46.2)	195	(48.3)
	HBS *- *B*-thalassaemia	2	(1.9)	4	(1.34)	6	(1.5)
	Not Available	46	(43.8)	157	(52.5)	203	(50.2)

* Sickle hemoglobin.

**Table 3:** Associations of socio-demographic characteristics with time to lost to follow-up (LTFU) among patients registered in a comprehensive sickle cell disease program led by an NGO in a tribal area of Gujarat, India during December 2015–June 2017; N = 404.

Characteristics	Categories	Person-years	LTFU	LTFU per 100 person-years	Unadjusted HR * (95% CI)	Adjusted HR * (95% CI)
Age in years	0–13	147.0	21	14.3	1	1
	14–29	313.2	73	23.3	1.6 (1.0–2.6)	0.7 (0.3–1.3)
	>30	73.2	18	24.6	1.8 (1.0–3.4)	0.5 (0.2–1.2)
Gender	Male	214.6	32	14.9	1	
	Female	318.8	80	25.1	1.7 (1.1–2.6)	1.3 (0.8–2.1)
Education	NFE **	48.0	21	43.8	1.8 (1.1–3.0)	1.4 (0.8–2.6)
	Primary	282.1	43	15.2	0.7 (0.4–1.0)	0.6 (0.4–0.9)
	More than primary	203.3	48	23.6	1	1
Occupation	Unemployed	211.7	55	26.0	1.8 (1.2–2.8)	0.8 (0.4–1.6)
	Employed	93.7	25	26.7	1.9 (1.1–3.1)	0.9 (0.4–1.9)
	Student	228	32	14.0	1	1
Marital Status	Married	194.0	66	34.0	2.4 (1.7–3.5)	3.2 (1.7–6.0)
	Unmarried	339.4	46	13.6	1	1
Caste	Scheduled tribe	529.3	111	21.0	0.85 (0.1–6.5)	-
	Backward classes	4.1	1	24.5	1	
Distance	<=20	248.3	34	13.7	1	1
	20–50	246.1	64	26.0	1.8 (1.2–2.7)	1.5 (1.0–2.4)
	>=51	39.0	14	35.9	2.2 (1.2–4.1)	1.2 (0.6–2.4)

* Hazard ratio. ** No-Formal education.

**Table 4:** Associations of clinical characteristics with time to lost to follow-up among patients registered in a comprehensive sickle cell disease program led by an NGO in a tribal area of Gujarat, India during December 2015–June 2017; N = 112.

Characteristics	Categories	Person-years	LTFU number	LTFU per 100 person-years	Unadjusted HR * (95% CI)	Adjusted HR * (95% CI)
Hospitalized at registration	Yes	403.8	71	31.6	1.7 (1.1–2.5)	1.7 (1.1–2.6)
	No	129.6	41	17.6	1	1
Number of pain crisis episodes in the last year	0	75.9	6	7.9	1	1
	1	86.9	23	26.5	3.1 (1.3–7.6)	3.0 (1.2–7.5)
	2	113.8	33	28.9	3.2 (1.4–7.7)	3.6 (1.5–8.8)
	>=3	256.7	50	19.5	2.1 (0.9–5.0)	2.4 (1.0–5.7)
Number of hospitalizations in the last year	0	349.5	72	20.6	1	1
	1	115.3	24	20.8	1.1(0.7–1.7)	-
	2	40.9	11	26.9	1.4 (0.8–2.7)	-
	>=3	27.8	5	18.0	1.0 (0.4–2.4)	-
Number of blood transfusions in the last year	0	423.9	91	21.4	1	1
	1	54.2	16	29.5	1.48 (0.9–2.5)	1.2 (0.7–2.1)
	>=2	55.3	5	0.90	0.5 (0.2–1.2)	0.3 (0.1–0.8)
Severe anemia	Yes	70.4	98	19.9	0.9 (0.5–1.6)	-
	No	463.0	14	21.2	1	
Presence of sickle cell complications	Yes	22.2	7	31.5	1.6 (0.8–3.5)	1.1 (0.5–2.6)
	No	511.2	105	20.5	1	1

* Hazard ratio.

**Table 5:** The coverage of clinical interventions and clinical outcomes among patients currently in care who registered between December 2015 and June 2017 in a comprehensive sickle cell disease program led by an NGO in a tribal area of Gujarat, India; N = 404.

Clinical Intervention *	Pre-registration, n	%	Post-registration, n	%
Received pneumococcal 23 vaccine	46	(11.4)	332	(82.2)
Prescribed daily dose of folic acid during last visit	358	(88.6)	380	(94.1)
Complete blood count done at least once in the last year	NA **	NA **	321	(79.5)
Liver function tests done at least once in the last year	NA **	NA **	245	(60.6)
Renal function tests done at least once in the last year	NA **	NA **	247	(61.1)
Initiated hydroxyurea to eligible patients (N = 137)	6	(4.4)	135	(98.5)
Prescribed hydroxyurea to eligible patients during last hospital visit (N = 137)	6	(4.4)	82	(59.9)
Pain crisis rate (per 100 person-years)	277	-	53.4	-
Hospitalizations rate (per 100 person-years)	49.8	-	42.2	-
Blood transfusion rate (per 100 person-years)	27.4	-	17.8	-
Hemoglobin level (g/dL) during last visit (mean (SD))	8.66	-	9.4	-
Diagnosed at least once with severe anemia *** the in last year, n (%)	61	(15.1)	16	(4.0)
Diagnosed with at least one sickle cell complication in the last year	16	(4.0)	31	(7.7)

* Interventions proven to be beneficial in sickle cell anemia care and adopted into the standard treatment guidelines of the comprehensive sickle cell clinic. ** NA = not available. *** Hemoglobin level less than 7 g/dL.

## References

[1] World Health Organization Sickle cell anaemia, report by secretariat. Proceedings of the Fifity-Ninth World Health Assembly.

[2] Upadhye D.S., Jain D.L., Trivedi Y.L., Nadkarni A.H., Ghosh K., Colah R.B. (2016). Neonatal screening and the clinical outcome in children with Sickle cell disease in central India. PLoS ONE.

[3] Dipty J., Khushnooma I., Vijaya S., Kanjaksha G., Roshan C. (2012). Sickle Cell Anemia from Central India: A Retrospective Analysis. INDIAN Pediatr..

[4] Piel F.B., Hay S.I., Gupta S., Weatherall D.J., Williams T.N. (2013). Global Burden of Sickle Cell Anaemia in Children under Five, 2010–2050: Modelling Based on Demographics, Excess Mortality, and Interventions. PLoS Med..

[5] Colah R., Mukherjee M., Ghosh K. (2014). Sickle cell disease in India. Curr. Opin. Hematol..

[6] Colah R.B., Mukherjee M.B., Martin S., Ghosh K. (2015). Sickle cell disease in tribal populations in India. Indian J. Med. Res..

[7] Pillay R. (2011). Census of India 2011: Provisional Population Totals, Chhattisga.

[8] Patel J., Patel B., Gamit N., Serjeant G.R. (2013). Screening for the sickle cell gene in Gujarat, India: A village-based model. J. Community Genet..

[9] Desai G., Dave K.K., Banerjee S., Babaria P., Gupta R. (2016). Initial outcomes of a comprehensive care model for sickle cell disease among a tribal population in rural western India. Int. J. Community Med. Public Heal..

[10] National Health Mission Maharastra Sickle Cell Disease Control Program. Proceedings of the 2nd National Summit on Good, Replicable Practices & Innovations in Public Healthcare Systems in India.

[11] Society for Education Welfare and Action-Rural (2015). Sickle Cell Comprehensive Care Programme.

[12] Commissionerate of Health Family Welfare and Medical Services (2016). Sickle Cell Anemia Program Manual.

[13] (2014). NHM, G. Sickle_Cell_Anemia_Control. https://nhm.gujarat.gov.in/sickle-cell.htm.

[14] Nimgaonkar V., Krishnamurti L., Prabhakar H., Menon N. (2014). Comprehensive integrated care for patients with sickle cell disease in a remote aboriginal tribal population in southern India. Pediatr. Blood Cancer.

